# ICD-11-Based Assessment of Social Media Use Disorder in Adolescents: Development and Validation of the Social Media Use Disorder Scale for Adolescents

**DOI:** 10.3389/fpsyt.2021.661483

**Published:** 2021-04-22

**Authors:** Kerstin Paschke, Maria Isabella Austermann, Rainer Thomasius

**Affiliations:** German Center for Addiction Research in Childhood and Adolescence, Deutsches Zentrum für Suchtfragen des Kindes- und Jugendalters (DZSKJ), University Medical Center Hamburg-Eppendorf (UKE), Hamburg, Germany

**Keywords:** problematic social media use, behavioral addictions, ICD-11, adolescents, validation, questionnaire, social media use disorder

## Abstract

**Background:** A problematic social media use (PSMU) in adolescents is a rising phenomenon often associated with higher perception of psychological stress and comorbid psychiatric disorders like depression. Since the ICD-11 introduced the very first internet-use related disorders, criteria for gaming (and online gambling) disorder can now be transferred to assess social media use disorder (SMUD). Therefore, the development and validation of a self-rating screening instrument for SMUD is of value to researchers and clinicians.

**Method:** The previously validated ICD-11-based Gaming Disorder Scale for Adolescents (GADIS-A) was adapted to measure SMUD (Social Media Use Disorder Scale for Adolescents, SOMEDIS-A). A representative sample of 931 adolescents aged 10 to 17 years and a respective parent participated in an online study. Item structure was evaluated by factorial analyses. Validated DSM-5-based instruments to assess PSMU by self- and parental ratings (SMDS, SMDS-P), adolescent depressive symptoms (PHQ-9), and stress perception (PSS-10) as well as single items on time spent with social media (SM, frequency and duration) were applied to assess criterion validity. Discrimination between pathological and non-pathological users was examined based on ROC analyses retrieved cut-off values and the results of a latent profile analysis.

**Results:** The new scale is best described by two factors reflecting cognitive-behavioral symptoms and associated negative consequences. The internal consistency was good to excellent. The SOMEDIS-A-sum score was positively correlated with PSMU, depression, and stress scores as well as the time spent with SM in a moderately to highly significant manner. Thus, good to excellent criterion validity is suggested.

**Conclusions:** SOMEDIS-A is the first successfully validated instrument to assess SMUD in adolescents based on the ICD-11 criteria of GD. Thus, it can support early detection in order to prevent symptom aggravation, chronification, and secondary comorbidities. It can contribute to the development of a standardized conceptualization and its two-factorial structure offers promising new insights into the evaluation of SM usage patterns. Further examination including clinical validation is desirable.

## Introduction

With access to fast and reliable internet increasingly available, social media (SM) applications are becoming an integral part of people's lives worldwide. According to a representative survey on German families, 93% of the 12- to 19 year-olds in Germany own a smartphone and rate SM apps (especially WhatsApp, Instagram, and YouTube) as their favorite internet services (1). Over 80% of these adolescents reported unrestricted internet access, allowing them almost unlimited SM usage. During the last years and especially since the beginning of the COVID-19 pandemic, adolescent smartphone usage has significantly increased (2). SM has become very important for staying in touch during a period of restrictive social interaction. This is supported by a representative study on 10- to 17-year-old adolescents who mainly used SM to fight boredom (86%), stay in contact with others (89%), and get information on the pandemic (37%) (3). Almost one third of these adolescents reported using SM to forget sorrows (38%), reduce stress (36%), and escape reality (36%). Interestingly, in a recent study on motivations for using social networking sites from late adolescence to early adulthood, Stockdale et al. identified the motives to socially connect and to fight boredom as risk factors for problematic SM use, in contrast to the motive information seeking (4).

There is an ongoing debate whether SM use can take on at-risk or even pathological dimensions, and consequently be described by the criteria for addictive disorders, since longitudinal studies for comparison with other addictions are missing (5–7). However, it has been repeatedly suggested to describe problematic usage patterns by the same set of diagnostic criteria as other addictive disorders (8, 9), including pathological gaming (10), due to similarities between the phenomena (5). In line with this, the aforementioned motives for using SM–*forgetting sorrows, reducing stress*, and *escaping reality* – are reminiscent of the *escape* criterion described in the context of internet gaming disorder (IGD) – the first digital media addiction included in a diagnostic manual (DSM-5) as a condition warranting more research (11). Accordingly, if five out of nine criteria have been met for the past 12 months, an IGD can be assumed. These criteria include *preoccupation, withdrawal* (when not using), *tolerance, persistence* (unsuccessful attempts to reduce or stop usage), *continuation* (of usage despite problems), *deception* (deceiving or covering up usage), *escape* (usage to avoid or reduce adverse moods), *displacement* (giving up other activities), and *conflict* (risking or losing relationships or career opportunities due to excessive usage). Furthermore, as the first official digital media-associated diagnoses, the gaming disorder (GD) and the (online) gambling disorder will be included in the 11th revision of the International Classification of Diseases (ICD-11), under the parent *Disorders due to substance use or addictive behaviors* (12). Both addictive behaviors are described by the following criteria concerning a continuous or episodical on-/off-line usage pattern that is generally present over a period of at least 12 months: (A) impaired control, (B) increasing priority over other activities, (C) continuation or escalation despite the occurrence of negative consequences and (D) the behavior results in clinically significant distress or impairment of personal, social, educational, work-related, and financial functions. Hence, in contrast to the DSM-5 criteria, both symptoms and significant impairments arising from these symptoms must be evident for the diagnoses to be met. In addition, the terms hazardous gaming (HG) and hazardous gambling (and betting) were introduced by the ICD-11, to describe distinct persistent behavioral patterns with awareness of increased risk of physical or psychological harm to self or others due to frequency and duration of use, neglect of alternative activities, risky usage-associated behaviors, and/or negative consequences (12). The results of studies comparing DSM-5- with ICD-11 criteria for pathological gaming suggest that DSM-5 criteria indicate a lower diagnostic threshold (13, 14), thus comprising at-risk and pathological behavior.

To date, no consensus has been found on nomenclature and definitions of concepts regarding problems associated with social media use. Terms include, e.g., *social media addiction* (15), *excessive social media use* (16), *social media dependence* (17), *social media disorder* (10), and *problematic social media use* (18, 19). Accordingly, there has been no agreement on the measurement, leading to conceptual and empirical ambiguity (5).

Most available scales were based on general criteria of addictive disorders (cf. six core components of addiction model by Griffith) (20) or the DSM-5 criteria for IGD [e.g., (10, 21–23)]. According to Billieux et al., the problematic use of SM may depend on a constellation of factors that are unique to this activity and not necessarily relevant when considering other types of internet addiction (24). Other available scales were therefore conceptualized based on literature review and/or expert interviews (25, 26). Interestingly, these show large content overlap with scales developed based on general addictive and IGD concepts (10, 15), thereby debunking assumptions of unrelated entities (27). To date, no scales have been developed based on the ICD-11 framework. We could identify only a few instruments that have explicitly assessed problematic use of SM and have been validated in samples of adolescents (10, 15, 25, 26). The 9-item Social Media Disorder Scale (SMDS) by van den Eijnden et al. (10) was developed based on the IGD criteria. It was validated in samples of adolescents in Europe and China (10, 22, 28, 29), thus making it one of the most widely used scales to assess a problematic use of SM in this age group.

Recently, the SMDS was used with 10- to 17-year old adolescents from 29 European countries to estimate prevalence rates for problematic SM use (23). The average prevalence was 7.38%, with country-specific values ranging from 3.22% (Netherlands) to 14.17% (Spain). Adolescents seem to be especially vulnerable for developing digital-media associated behavioral addictions (30). On the one hand, they are attracted by the structure and the design of digital apps that built upon psychological mechanisms to achieve strong attachment and increase the time spent with them (31). On the other hand, adolescents' cognitive control abilities contrast with fully developed reward systems leading to an increased sensitivity to motivational cues [cf. neurobiological imbalance model of adolescence, (32)].

Problematic SM use positively correlates with the time spent with SM (8, 33, 34). It is often associated with symptoms of psychiatric disorders like depression, anxiety disorders, attention deficit hyperactivity disorder, obsessive compulsive disorder, and eating disorders (35–37). Moreover, affected adolescents report sleep deprivation with negative effects on daily functioning and mood, lower emotional well-being, higher general stress, as well as stress related to peer neglect (35, 38, 39). As a result, problematic SM use has become a concern for healthcare professionals in recent years. It is currently unclear whether the observed increased time spent with SM use during the COVID-19 pandemic will result in a higher prevalence of problematic use in adolescents over time. Given the increased use and the fact that SM have become part of everyday life, it is particularly important not to exaggerate or globally pathologize intensive patterns of use (24, 40), but rather to detect individual behaviors that might need intervention. Since the ICD-11 criteria require both specific symptoms and significant impairments arising from these symptoms to be present for a potential diagnosis, a higher specificity to detect pathological users can be assumed. As the first ICD-11 GD screening tool for adolescents, the two-factorial Gaming Disorder Scale for Adolescents (GADIS-A) was published by the authors in 2020 (41). It had revealed good to excellent internal consistency, validity, and discriminatory power. The items covered the factors cognitive-behavioral GD symptoms and negative consequences as well as a time criterion. This makes it an interesting candidate for modifying the ICD-11 framework to assess pathological SM use in adolescents.

We will use the term *problematic social media use* (PSMU) in this manuscript to refer to at-risk and pathological SM use based on the DSM-5 criteria for IGD. In addition, analog to the term GD to describe pathological gaming according to the ICD-11 framework, we will use the term *social media use disorder* (SMUD) to describe pathological SM use that potentially requires therapeutic treatment. SMUD is thereby delimited from the term PSMU by its higher specificity.

To the best of our knowledge, despite the potential value to clinicians and researchers working with minors, no validated ICD-11-based screening instrument for SMUD in adolescents is available at this point. Therefore, the aims of this study were (1) the development of a SMUD screening instrument for adolescents (Social Media Use Disorder Scale for Adolescents, SOMEDIS-A) by adapting GADIS-A, (2) the exploration of the psychometric properties of the newly developed scale, and (3) in line with the validation of the original scale, its validation in a representative sample of 10- to 17-year-old frequent (SM) users and a respective parent.

## Method

### Participants and Procedures

14,472 randomly selected German households with adults aged 28 to 75 years were invited by email to participate in an online survey on family media use between November 10 and December 01, 2020. The invited households belong to a continuously growing panel of currently ~75,000 randomly selected adults and adolescents aged 14 years and above (42). Of 6,764 respondents, 726 reported having children between the ages of 10 and 17 years. Of these, 557 parents and one child each provided necessary information and gave their informed consent to participate in the survey. Additionally, 1,221 representative households that had participated in a previous representative survey were contacted in the same period. Of these, 585 had a child in the age of interest and agreed to take part. This led to a total number of *N* = 1,142 participating parent-child dyads. Representativity was ensured regarding region of residence, age, and gender of the participants by the established German market research and opinion polling company forsa based on a random sampling method (42) [for details on the recruitment and sampling method see Paschke et al. (43)]. Parents and adolescents were asked to complete the questionnaires independently after one another.

The study was conducted in accordance with the relevant national and institutional committees on human experimentation, complied with the Declaration of Helsinki, and was approved by the Local Psychological Ethics Commission at the Center for Psychosocial Medicine (LPEK) of the University Medical Center Hamburg-Eppendorf (UKE). Participants could withdraw from the study at any time, for any reason.

1,041 out of 1,142 adolescents [92.0%, 530 boys (50.9%) and 511 girls (49.1%)] reported a SM use of at least once a week and were, together with the corresponding parent, considered for further statistical processing. Of these, 110 had to be excluded due to missing data of more than one third per scale, resulting in a final sample of *N* = 931 parent-child dyads.

### Measures

#### Social Media Usage Patterns

In the online survey, SM were defined as all digital services on which texts, photos, animations, or videos can be shared, commented on or liked (e.g., Instagram, TikTok, YouTube). SMUD was assessed based on the ICD-11 criteria of Gaming Disorder by the newly developed Social Media Use Disorder Scale for Adolescence (SOMEDIS-A). SOMEDIS-A was adapted from the validated ICD-11 based Gaming Disorder Scale for Adolescents (GADIS-A) (41) by clinical experts and scientists in the field of behavioral addictions in adolescence. Thinking of the last 12 months, the adolescents were asked to state their agreement with nine statements choosing one out of five (Likert-scale) response options (strongly disagree−0, somewhat disagree−1, partially agree/ partially disagree−2, somewhat agree−3, strongly agree−4). These could be summed up to a maximum score of 36. The frequency and duration of problems, conflicts, or difficulties due to SM use was assessed by an additional question with four response options (not at all—, only on single days−1, for longer periods−2, nearly daily−3). A score of 2 and above was considered significant regarding the ICD-11-time criterion. The GD adapted ICD-11 symptoms and their corresponding DSM-5 criteria are displayed in Table 1 together with the English version of the SOMEDIS-A items. Symptoms A to C were covered by two items each. Impairment (D) was addressed by three items on personal, social and educational/working difficulties caused by SM use. The complete questionnaire can be found in the Appendix.

**Table 1:** SOMEDIS-A items with corresponding ICD-11 and DSM-5 criteria.

**ICD-11 criteria^**a**^ and corresponding *DSM-5* item**	**SOMEDIS-A items**
	**Thinking of the last 12 months, how strongly do you agree with the following statements?**
A) Impaired control over SM use (e.g., onset, frequency, intensity, duration, termination, context)	1. I often use social media more frequently and longer than I planned to or agreed upon with my parents^b^
*Persistence*	2. I often cannot stop using social media even though it would be sensible to do so or for example my parents have told me to stop
B) Increasing priority given to SM use to the extent that it takes precedence over other life interests and daily activities	3. I often do not pursue interests outside the digital world (e.g., meeting friends or partner in real life, attending sports club/societies, reading books, making music) because I prefer using social media
*Displacement*	4. I neglect daily duties (e.g., grocery shopping, cleaning, tidying up after myself, tidying my room, obligations for school/apprenticeship/job) because I prefer using social media
C) Continuation or escalation of SM use despite the occurrence of negative consequences	5. I often continue using social media even though it causes me stress with others (e.g., my parents, siblings, friends, partner, teachers)
*Continuation*	6. I continue using social media although it harms my performance at school/apprenticeship/job (e.g., by being late, not participating in class, neglecting homework, worse grades)
D) The behavioral pattern is of sufficient severity to result in significant impairment in personal, family, social, educational, occupational, or other important areas of functioning *Conflict*	7. Due to my social media use, I neglect my appearance, my personal hygiene, and/or my health (e.g., sleep, nutrition, exercise) 8. Due to my social media use, I risk losing important relationships (friends, family, partner) or have lost them already 9. Due to my social media use, I have disadvantages at school/apprenticeship/job [e.g., bad (final) grades, inability to continue to the next grade/no graduation, no apprenticeship or university spot, poor reference, warning/dismissal]
E) The pattern of SM use may be continuous or episodic and recurrent and normally evident over a period of at least 12 months	10. How often did you experience such problems, conflicts, or difficulties due to social media use during the past year? Did this only occur on single days, during longer periods of several days to weeks or months, or was it almost daily?^c^

SOMEDIS-A, Social Media Disorder Scale for Adolescents; ICD-11, 11th revision of the International Classification of Diseases; DSM-5, 5th revision of the Diagnostic and Statistical Manual of Mental Disorders; SM, social media;

a ICD-11 Gaming Disorder criteria adapted to social media usage;

b response options for item 1-9: 5-point Likert-Scale: “strongly disagree”- “strongly agree”;

c response options: “not at all,” “only on single days,” “during longer periods,” “almost daily.”

To compare the results of the new scale with validated DSM-5 based scales, PSMU was assessed by the Social Media Disorder Scale in its self- (SMDS) (8) and parental-judgement version (SMDS-P) (44). The SMDS was developed based on the DSM-5 criteria for IGD and the Internet Gaming Disorder Scale (IGDS) (45). A higher total score of the one-factorial polythetic questionnaire including nine items with a dichotomous response format (no—0/yes—1) indicated a higher risk for PSMU. The SMDS had been repeatedly applied to adolescent samples and showed adequate to good psychometric properties (8–10). Its parental version was validated in a representative sample of German adolescents and their parents to add external views and revealed good psychometric properties (44). In the sample of the current study both scales showed a good internal consistency (SMDS: Cronbach's α = 0.81; SMDS-P: Cronbach's α = 0.85). Analog to the procedure of Ko et al. (13) and Jo et al. (14) for IGD, SMDS items reflecting the DSM-5 criteria that correspond to the ICD-11 (*persistence, displacement, problem, conflict*) were considered separately.

PSMU has been found to positively correlate with the time spent with SM (8, 33, 34). The temporal pattern of SM use was measured by querying the average number of usage days per week (frequency) as well as the average usage duration on week (school) days and on weekend (leisure) days. Out of the two measures a mean daily usage time was calculated.

#### Psychological Stress Perception and Depressive Symptoms

Psychological stress and depressive symptoms were shown to be associated with PSMU (35, 36, 39). Therefore, these constructs were included to assess additional criteria validity. The level of psychological stress was determined by the Perceived Stress Scale (PSS-10) (46) — a 10-item self-report scale that has been validated in adolescents (47). They were asked to rate the frequency of statement contents within the past month on a five-point Likert scale (never—1 to always—5 for negatively, and inversed for positively phrased items). Higher scores indicated higher stress perception. The internal consistency of the scale in the current sample was good (Cronbach's α = 0.82). The 9-item Patient Health Questionnaire (PHQ-9) was used to assess depressive symptoms on a 4-point Likert scale in the adolescents (agreement to given statements: not at all—0 to nearly every day—3). It was originally described by Kroenke, Spitzer, and Williams (48) based on the DSM-IV and has been modified for adolescent samples (49, 50). The word “dead” in the last item (“thoughts that you would be better off dead, or of hurting yourself in some way”) was exchanged by “gone” to be more suitable for the anonymous online survey assessment that does not enable personal contact with the interviewee. The internal consistency of the scale in this study was also good (Cronbach's α = 0.88).

### Statistical Analyses

#### Data Management

Missing values of the final sample were replaced by performing multiple imputations in the statistical program R using the package mice (51, 52). This led to a total replacement of 0.18% (SOMEDIS-A), 1.31% (SMDS), 2.92% (SMDS-P), 0.55% (PHQ-9), and 1.45% (PSS-10) per instrument. The data was revised for normality distribution if appropriate. Absolute values of skewness >2.0 and kurtosis >7.0 served as reference values to determine substantial univariate non-normality (53). Of all scale variables, this was the case for the individual SOMEDIS-A item 8 (skewness = 2.3, kurtosis = 5.63) and item 9 (skewness = 2.02, kurtosis = 4.16). Multivariate normality was investigated by Mardia's test using the R package QuantPsyc (54).

#### Factor Analyses

A split-half validation method was applied before conducting an exploratory factor analysis (EFA) and a confirmatory factor analysis (CFA) with diagonally weighted least squares (DWLS) to account for the ordinal variable structure and multivariate non-normality using the R packages psych and lavaan (55, 56). For this purpose, the sample was randomly divided into two (nearly) equal proportions by the R package rsample (n_1_ = 466 dyads; n_2_ = 465 dyads) (57). The Kaiser-Meyer-Olkin (KMO) criterion and Bartlett's test of sphericity were calculated to affirm the suitability of the data for factor analysis. The visual scree test, parallel analysis, and the Wayne Velicer's Minimum Average Partial (MAP) criterion were applied to reveal the appropriate number of factors following the recommendation of Velicer et al. (58). The authors state that for sample sizes ≥ 300 a minimum ratio of 4:1 variables per factor leads to an accurate determination of factors (58). CFA model goodness of fit was assumed according to the following criteria: χ^2^/df ratio <5, root mean square error of approximation (RMSEA) <0.08, standardized root mean squared residual (SRMR) <0.08, Tucker-Lewis Index (TLI) ≥ 0.95, comparative fit index (CFI) ≥ 0.95 (59). The Satorra-Bentler mean adjusted χ^2^-difference statistic was used to compare model fits (60).

#### Internal Consistency

The coefficients Cronbach's α and McDonald's ω were calculated to determine internal consistency with the following interpretation: ≥0.9–excellent, ≥0.8–good, ≥0.7–acceptable, ≥0.6–questionable, ≥0.5–poor, and <0.5–unacceptable (61, 62).

#### Criterion Validity

Depending on the item/scale distribution, Pearson and Spearman rank correlations between the SOMEDIS-A sum score and the total scores of the questionnaires SMDS, SMDS-P, PHQ, and PSS-10 as well as the mean time spent with SM per day (in minutes) and the usage days per week were computed to obtain criterion validity based on the following interpretation: 0.00 ≤ Pearson's *r* ≤ 0.10 zero or negligible relationship; 0.10 < *r* ≤ 0.30 weak relationship; 0.30 < *r* ≤ 0.50 moderate relationship; r > 0.5 strong relationship (63); 0 ≤ Spearman's ϱ ≤ 0.10 zero or negligible relationship; 0.1 < ϱ ≤ 0.40 weak relationship; 0.40 < ϱ ≤ 0.70 moderate relationship; 0.70 < ϱ ≤ 0.90 strong relationship; ϱ > 0.90 perfect relationship (64).

#### Sensitivity and Specificity

Sensitivity and specificity across SOMEDIS-A sum scores were compared by a receiver operating characteristic (ROC) curve analysis to predict SMUD according to the SMDS classification. The analysis was realized using the R package pROC (65). 95% confidence intervals (CI) were estimated based on 999 bootstrapping replications. Youden's criterion was applied to define cut-off points. The area under curve value (AUV) reflected the goodness of differentiation between the two groups (66). Based on the calculated cut-off points, adolescents were classified as pathological or non-pathological SM users. Associated prevalence was estimated using 95% CI. The means and standard error of means (se) of age and SMDS, SMDS-P, PHQ, and PSS-10 sum scores as well as SM usage days per week and mean SM usage hours per day were calculated for each group. The variables were included in a MANOVA with *post-hoc* Scheffé tests to compare both groups. Given the large sample size, the central limit theorem applied and MANOVA test result could be assumed to be robust even though the assumption of multivariate normality was violated (67, 68). The proportion of sex of both groups was computed together with 95% CI and compared via χ^2^ test. Corresponding effect sizes were interpreted as follows: Cramer's V (categorial variables) >0.5 strong, >0.3 moderate, >0.1 weak effect (69); Cohen's d (metric variables) >0.8 large, >0.5 medium, >0.2 small effect (70).

#### Classification

In addition to a cut-off based classification, a latent-profile analysis (LPA) on the SOMEDIS-A factor sum scores and the SOMEDIS-A time criterion was performed to estimate the number of latent subgroups of SM users within the sample using the R package mclust (71). This package uses a model-based approach where each component of a Gaussian finite mixture density is associated with a profile. Scrucca et al. provide a detailed description of the underlying procedure and emphasize an appropriate application on data sets of various disciplines including clinical psychology (71). The adolescents' membership to a profile was inferred. Due to multivariate non-normality, the robustness of the LPA results was assessed by means of 999 non-parametric bootstrapping operations. Based on the results of the bootstrap likelihood ratio test (BLRT), the Akaike information criterion (AIC), the Bayesian information criterion (BIC), and the integrated completed likelihood (ICL), the ideal number of profiles was determined. The BLRT compared the fit between a model of a certain number of profiles and a model with one profile less (72). Bootstrap samples were used to estimate the distribution of the log likelihood difference test statistic. According to the null hypothesis, the smaller model was the best model. If the larger model fitted the data significantly better (*p* < 0.001), the null hypothesis would be rejected. Furthermore, lower BIC, AIC, and ICL values reflected better model solutions (73, 74). All profile groups were described regarding prevalence and sex by frequency estimations with 95% CI, as well as SOMEDIS-A factor 1, factor 2, and time criterion scores, age, SMDS, SMDS-P, PHQ, and PSS-10 sum scores, SM days per week, SM hours per day by means with standard error of means (se). The group proportions of sex were compared by χ^2^ test and the group differences regarding the SOMEDIS-A factors by effect-size estimation. The other dependent variables were included in a MANOVA with the latent profile group as independent factor and further evaluated by *post-hoc* Scheffé tests and effect-size estimation. Again, given the multivariate non-normality of the data, model robustness was assumed based on the central limit theorem.

## Results

### Sample Description

A detailed description of the final sample can be found in Table 2.

**Table 2:** Characteristics of final sample parent-child dyads^a^.

**Variables/categories**	**Adolescents *N* [% (95%–CI)]/mean (SD; range)**	**Parents *N* [% (95%–CI)]/mean (SD; range)**
Absolute frequency	931	931
**Gender**		
Male	468 [50.3 (47.1–53.5)]	466 [49.9 (46.7–53.2)]
Female	463 [49.7 (46.5–52.9)]	465 [50.1 (46.7–53.2)]
Age in years	13.67 (2.19; 10–17)	47.13 (7.62; 28–75)
**Relationship status**		
Biological child	851 [91.5 (89.7–93.3)]	
Adoptive child	6 [0.7 (0.1–1.2)]	
Stepchild	46 [5.0 (3.6–6.3)]	
Other^b^^,^ ^c^	27 [2.9 (1.8–4.0)]	
**Education level^d^^,^^e^**		
High	291 [60.9 (56.5–65.3)]	285 [30.7 (27.7–33.7)]
Medium	150 [31.4 (27.2–35.5)]	548 [59.1 (55.9–62.2)]
Low	37 [7.7 (5.3–10.1)]	95 [10.2 (8.3–12.2)]
**Occupation**^***f***^		
Full-Time employment/school attendance	415 [86.5 (83.4–89.5)]	570 [61.4 (58.2–64.5)]
Part-Time employment/apprenticeship	43 [9.0 (6.4–11.5)]	255 [27.5 (24.6–30.3)]
Other^g^	22 [4.5 (0.5–8.7)]	104 [11.4 (8.0–14.4)]
**Place of residence**		
Urban living^h^	766 [17.7 (15.3–20.2)]	
Rural living	165 [82.3 (79.8–84.7)]	
**Psychological measures**		
PSS-10 sum score	15.19 (6.63, 0–39)	–
PHQ-9 sum score	4.50 (4.52, 0–27)	–
SMDS/SMDS-P sm score	1.54 (2.08, 0–9)	1.71 (2.36, 0–9)

N, absolute frequency; CI, confidence interval; SD, standard deviation; PSS-10, Perceived Stress Scale; PHQ, Patient Health Questionnaire; SMDS-(P), Social Media Disorder Scale (Parental Version);

a dyads with frequently social media using adolescents, i.e., adolescents use social media at least once a week;

b foster child/not specified;

c no response n = 1;

d for parents: highest level achieved–high = bachelor/master's degree to doctorate (Ph.D), medium = secondary school-leaving certificate (Realschulabschluss)/university entry qualification (Abitur)/completed apprenticeship, low = no or lower school-leaving certificate (Hauptschulabschluss); for adolescents: (prospective) school leaving certificate (based on the current school performance)–high = university entry qualification (Abitur), medium = secondary school certificate (Realschulabschluss), low = no/special-school (Förderschulabschluss)/lower school certificate (Hauptschulabschluss);

e no response adolescents n = 453, no response parents n = 3; no response/item not presented to adolescents younger than 14 years;

f no response adolescents n = 451, no response parents n = 2; no response adolescents/item not presented to adolescents younger than 14 years;

g for adolescents: university students, in voluntary service, military service, other occupation, or unemployed; for parents: job-seeking, welfare recipient, pensioners, disabled, trainee, student, no specification;

h *areas with ≥ 5,000 residents*.

### Factor Structure

Bartlett's test revealed significant correlations between the nine SOMEDIS-A items on the first half of the sample data [χ^2^(34) = 2,402.13, *p* < 0.001]. KMO criterion was 0.88 overall for the first sub-sample and ranged between 0.83 and 0.95 for individual items. Thus, good suitability of the data for EFA could be demonstrated (75). Visual scree test, parallel analysis, and MAP criterion suggested that two factors should be retained (eigenvalue factor 1 = 5.13 and eigenvalue factor 2 = 1.14; minimum Velicer MAP of 0.05). Communalities of the individual items ranged from 0.50 to 0.76. The cumulative variance explained by the two factors was 0.62 (variance of factor 1 = 0.35). Factor loadings varied between 0.59 and 0.82 for factor 1 and 0.56 and 0.84 for factor 2. A CFA based on a 2-factorial model yielded mixed results: On the one hand, CFI of 0.993 and TLI of 0.990 indicated excellent fit and SRMR of 0.058 as well as χ^2^/df ratio of 4.96 [χ^2^(23) = 129.04, *p* < 0.001] acceptable fit. On the other hand, RMSEA of 0.092 indicated a poor fit. Yet, a two-factorial model suggested a significantly better fit to the data than a single-factor solution [$\chi_{diff}^{2}$ (1) = 52.29, *p* < 0.001]. All item factor loadings were significantly positive, with standardized coefficients lying between 0.73 and 0.90.

**Figure 1:**
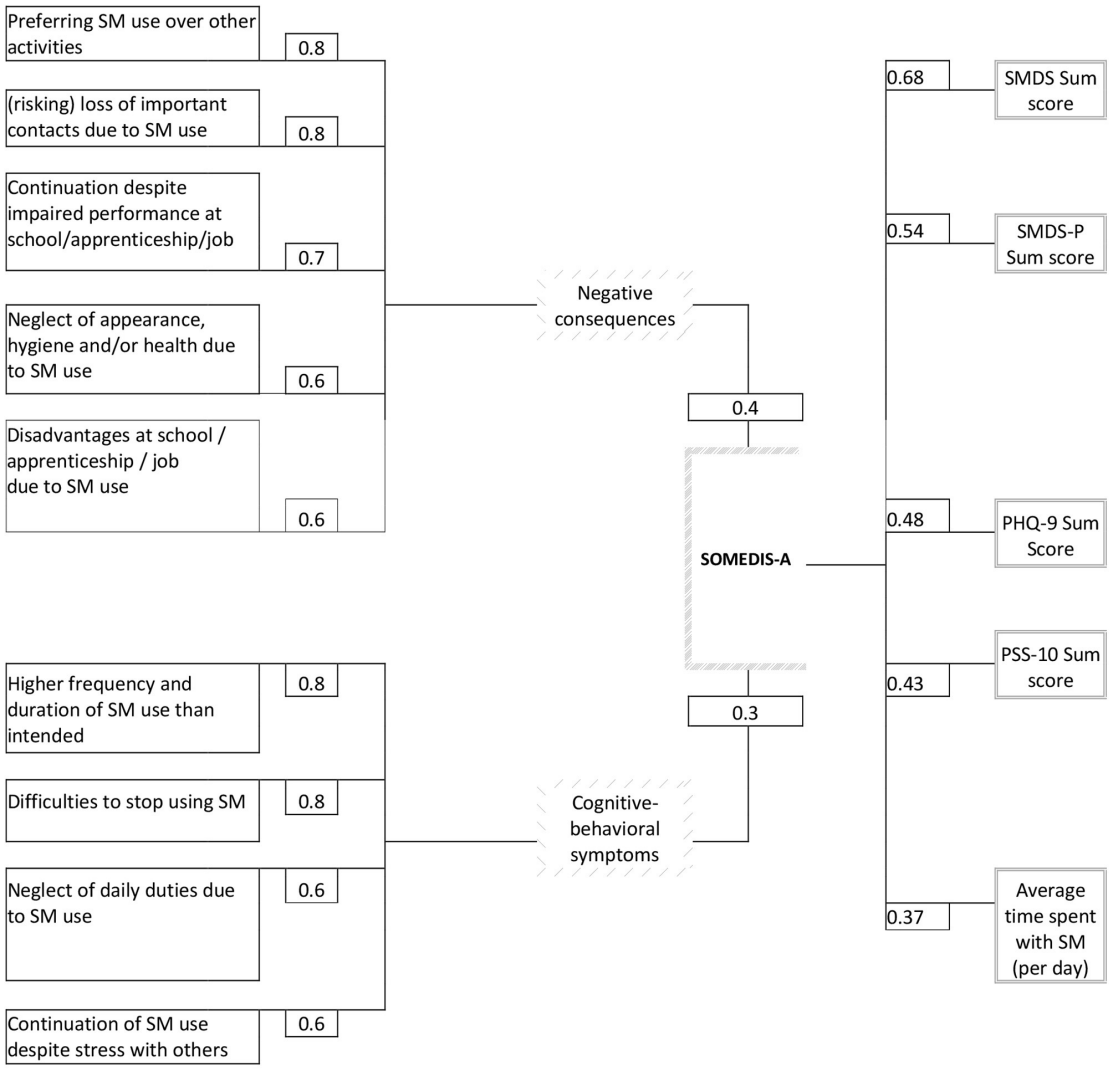
EFA factor loadings on the latent SOMEDIS-A factor 1 (negative consequences) and SOMEDIS-A factor 2 (cognitive-behavioral SM use symptoms) are shown on the left [next to the individual scale item (manifest variable)] together with the proportion of explained variance (given above and below the SOMEDIS-A box). Correlation coefficients of the SOMEDIS-A sum score with criteria are presented on the right side (next to the criteria variables). All factor loadings and correlations were significant with p < 0.001. The usage days per week did not significantly correlate with the SOMEDIS sum score and are, thus, not depicted. SOMEDIS-A, Social Media Disorder Scale for Adolescents; SMDS(-P), Social Media Disorder Scale (Parental Version); SM, social media; PHQ, Patient Health Questionnaire; PSS, Perceived Stress Scale.

SOMEDIS-A items 7 to 9 (personal, social, and academic/occupational impairments), 6 (continuation despite academic/occupational disadvantages), and 3 (loss of other interests due to gaming) loaded highest on factor 1. This factor reflects impending or manifest consequences due to SM use. SOMEDIS-A items 1 and 2 (loss of control), 5 (continuation despite social stress) and 4 (neglecting daily duties) loaded highest on factor 2 which symbolizes cognitive-behavioral symptoms associated with SM use. Figure 1 shows EFA-factor loadings and the variance proportion explained by the two factors. All EFA- and CFA- (standardized) factor loadings are presented in Table 3 together with the EFA communalities. Inter-item correlations and the relative item-response frequencies are depicted in Tables 4, 5. All items showed a moderate correlation with the time criterion (0.41 ≤ *r* ≤ 0.64).

**Table 3:** Factorial analyses of SOMEDIS-A items.

**SOMEDIS-A item^***a***^**	**Factor 1^***b***^**	**Factor 2^***b***^**	**Communalities**
Item 1 EFA	0.17	0.75	0.60
CFA	–	0.73	–
Item 2 EFA	0.26	0.84	0.77
CFA	–	0.86	–
Item 3 EFA	0.59	0.43	0.54
CFA	0.84	–	–
Item 4 EFA	0.50	0.57	0.58
CFA	–	0.82	–
Item 5 EFA	0.53	0.56	0.59
CFA	–	0.83	–
Item 6 EFA	0.70	0.38	0.63
CFA	0.90	–	–
Item 7 EFA	0.64	0.30	0.50
CFA	0.74	–	–
Item 8 EFA	0.78	0.18	0.63
CFA	0.86	–	–
Item 9 EFA	0.82	0.19	0.71
CFA	0.90	–	–

SOMEDIS-A, Social Media Disorder Scale for Adolescents; EFA, Explanatory Factor Analysis (based on split-half sub-sample of n1 = 466 dyads); CFA, Confirmatory Factor Analysis (based on split-half sub-sample of n2 = 465 dyads), SOMEDIS-A factor 1 = negative consequences, SOMEDIS-A factor 2 = cognitive-behavioral symptoms;

a *for the description of the items, refer to Table 1*.

b *(standardized) factor loadings are depicted*.

**Table 4:** Inter-item correlation of SOMEDIS-A items^a^.

**Items^***b***^**	**Item 1**	**Item 2**	**Item 3**	**Item 4**	**Item 5**	**Item 6**	**Item 7**	**Item 8**	**Item 9**	**Timing item**
Item 1	1.00									
Item 2	0.69	1.00								
Item 3	0.40	0.52	1.00							
Item 4	0.48	0.58	0.55	1.00						
Item 5	0.50	0.60	0.57	0.55	1.00					
Item 6	0.39	0.49	0.57	0.61	0.57	1.00				
Item 7	0.35	0.42	0.51	0.50	0.47	0.53	1.00			
Item 8	0.29	0.39	0.64	0.46	0.54	0.56	0.57	1.00		
Item 9	0.32	0.43	0.54	0.52	0.52	0.74	0.58	0.66	1.00	
Timing item	0.64	0.61	0.46	0.51	0.50	0.49	0.45	0.41	0.46	1.00

SOMEDIS-A, Social Media Disorder Scale for Adolescents;

a based on total sample of N = 931 adolescents;

b *for the description of items, refer to Table 1. The items of factor 2 are highlighted in gray*.

**Table 5:** Relative item-response frequency of SOMEDIS-A items (in %)^a^.

**SOMEDIS-A items^***b***^**	**Response options**				
	**Strongly disagree**	**Somewhat disagree**	**Partially agree/partially disagree**	**Somewhat agree**	**Strongly agree**
Item 1	18.4	23.6	31.6	19.3	7.1
Item 2	28.9	28.6	23.6	13.5	5.0
Item 3	61.0	23.2	9.9	4.3	1.6
Item 4	40.4	28.2	19.4	7.6	4.3
Item 5	48.4	26.7	15.8	6.4	2.6
Item 6	59.4	23.1	11.1	4.3	2.1
Item 7	61.5	23.3	9.9	3.3	1.9
Item 8	72.4	18.6	5.8	1.9	1.3
Item 9	68.1	20.9	7.3	2.3	1.4
	**Not at all**	**Only on single days**	**For longer periods**	**Nearly daily**	
timing item	35.3	58.3	4.1	2.3	

SOMEDIS-A, Social Media Disorder Scale for Adolescents;

a based on the total sample of N = 931 adolescents;

b *for the description of items, refer to Table 1. The items of factor 2 are highlighted in gray*.

### Internal Consistency

Regarding the total SOMEDIS-A scale, Cronbach's α of 0.91 and McDonald's ω of 0.93 were calculated. For the first factor-associated subscale, Cronbach's α of 0.88 and McDonald's ω of 0.91 were computed. For the second subscale, Cronbach's α was 0.84 and McDonald's ω was 0.86. Thus, the total scale indicates excellent and the two subscales good internal consistency.

### Criterion Validity

Strong positive correlations were found between the total sum scores of SOMEDIS-A and SMDS (Pearson's *r* = 0.68, *p* < 0.001) as well as SMDS-P (*r* = 0.54, *p* < 0.001). PSS-10 (*r* = 0.43, *p* < 0.001) and PHQ-9 (*r* = 0.48, *p* < 0.001) sum scores positively correlated with SOMEDIS-A sum score in a moderate manner. Whereas, the correlations between the SOMEDIS-A sum score and the average daily duration of SM usage was also moderately positive (*r* = 0.37, *p* < 0.001), no significant association could be found with the usage days per week (Spearman's ϱ = 0.05, *p* = 0.10). All significant coefficients are shown in Figure 1 (right column).

### Sensitivity and Specificity

Adolescents were classified as pathological or non-pathological SM users according to their responses on the ICD-11 related SMDS items. This classification was included into two ROC curve analyses together with the two SOMEDIS-A subscale sum scores (following the two-factorial scale structure). According to Youden's criterion, the optimal cut-off for SOMEDIS-A factor 1 was 6.5 (95% CI 6.5, 7.5) with a specificity of 87.39% (95% CI 87.39, 94.03), a sensitivity of 81.48% (95% CI 66.67, 96.30), an AUC value of 88.4% (95% CI 79.7, 97.0) and an accuracy of 89.47%. An optimal cut-off of 8.5 (95% CI 7.5, 9.5) was calculated for factor 2 with a specificity of 82.96% (95% CI 74.89, 91.04), a sensitivity of 88.89% (95% CI 74.07, 1.00), an AUC value of 88.8% (95% CI 80.1, 97.5) and an accuracy of 84.0%. Considering both factor cut-off values a good differentiation between adolescents with and without SMUD was indicated.

### Classification by Cut-Off Values

Applying the cut-off of >6 for factor 1 and >8 for factor 2 as well as considering the ICD-11-time criterion (symptoms at least for longer periods or daily), 3.3% (95% CI 2.2, 4.5) of the adolescent SM users could be classified as pathological (N = 31). Except for age, all ten dependent variables included in a MANOVA reached significance regarding adolescents with and without SMUD [Pillai score (1, 905) = 0.38, *F* (10, 896) = 55.14, *p* < 0.001]. Table 6 shows the MANOVA results and the comparison of affected and non-affected adolescents regarding sex as well as the variables included in the *post-hoc* MANOVA tests. No differences were found between the proportion of sex, age and number of usage days in either group. Pathological SM users showed (per definition) higher SOMEDIS-A subscale and time criterion scores, but also higher SMDS and SMDS-P as well as higher PHQ-9 and PSS-10 sum scores with a large effect sizes compared to uncritical SM users.

**Table 6:** MANOVA and *post-hoc* test results on adolescents ROC-classified with/without SMUD.

**Variables**	**SMUD**	**No SMUD**	***F*-value**	***χ^2^*/*post-hoc* Scheffé tests**	**Cramer's V/Cohen's d**
Absolute frequency	31	900	–	–	–
Relative frequency in % (95%–CI)	3.33 (2.18, 4.48)	96.67 (95.52, 97.82)	–	–	–
Female sex in % (95%–CI)	58.06 (40.69, 75.44)	49.44 (46.18, 52.71)	–	0.01 NS (*p* = 0.93)	–
Mean age (SE)	13.71 (0.29)	13.67 (0.07)	0.01 NS (*p* = 0.94)	–	–
Mean SOMEDIS-A factor 1 score (SE)	13.13 (0.88)	2.43 (0.1)	357.52^***^	10.7^***^	3.42
Mean SOMEDIS-A factor 2 score (SE)	12.45 (0.49)	4.81 (0.12)	146.06^***^	7.64^***^	2.19
Mean SOMEDIS-A time criterion score (SE)	2.42 (0.09)	0.67 (0.02)	294.82^***^	1.74^***^	3.11
Mean SMDS sum score (SE)	6.29 (0.44)	1.38 (0.06)	200.38^***^	4.91^***^	2.6
Mean SMDS-P sum score (SE)	5.71 (0.57)	1.58 (0.07)	104.14^***^	4.13^***^	1.84
Mean usage days per week (SE)	6.39 (0.29)	6.33 (0.05)	0.02 NS (*p* = 0.88)	–	–
Mean time spent with SM per day [in minutes] (SE)	337.77 (48.7)	159.46 (21.64)	57.79^***^	187.32^***^	1.39
PHQ sum score	12.71 (0.25)	4.21 (0.14)	118.9^***^	8.5^***^	2
PSS sum score	23.39 (0.18)	14.91 (0.22)	51.08^***^	8.5^***^	1.31

*** *p ≤ 0.001, NS, not significant; MANOVA, Multivariate Analysis of Variance; ROC, receiver operating characteristic; SMUD, social media use disorder; χ^2^, chi-square; Cramér's V/Cohen's d, effect sizes; (95%–CI), 95% confidence interval; SE, standard error of the mean; SOMEDIS-A, ICD-11 Social Media Disorder Scale for Adolescents; SMDS(-P), DSM-5 Social Media Disorder Scale (parental version); SOMEDIS-A factor 1, negative consequences; SOMEDIS-A factor 2, cognitive-behavioral symptoms; SM, social media; PHQ, Patient Health Questionnaire; PSS, Perceived Stress Scale*.

### Classification by LPA

A latent profile analysis (LPA) on the two SOMEDIS-A subscale and the time criterion score with an ellipsoidal, equal volume and shape model describing three profiles showed the best fit based on smallest AIC, absolute BIC, and ICL values (see Table 7). The log likelihood value was significantly smaller for a three-profile compared to a four-profile solution. Thus, including more profiles in the model did not suggest any benefit according to the likelihood ratio test (LRT). Robustness of this three-profile model could be shown by the bootstrapping procedure. Accordingly, the current sample could be divided into three mutually exclusive and exhaustive latent profiles that mirror SM usage patterns (based on the three SOMEDIS-A scores) as unobserved categorical variable.

**Table 7:** Comparison of number of latent classes according to latent profile analysis (LPA).

**Latent classes**	**Log likelihood**	**AIC**	**BIC**	**ICL**	**LRTS**
1	−3,342.78	6,703.56	−6,747.09	−6,747.09	0.00^***^
2	−3,243.62	6,519.23	−6,596.61	−6,600.03	198.33^***^
3	−2,293.1	4,632.2	−4,743.43	−4,743.44	1,901.04^***^
4	−2,293.16	4,646.33	−4,791.41	−5,005.09	−0.13

*** *p ≤ 0.001; LPA, Latent Profile Analysis; AIC, Akaike information criterion; BIC, Bayesian information criterion; ICL, Integrated Completed Likelihood; LRTS, likelihood ratio test score based on bootstrapping with 999 replications*.

More than half of the frequent SM users was classified in profile 2 based on the LPA results (*N*_*profile*2_ = 543; 58.3%), about one third in profile 3 (*N*_*profile*3_ = 329; 35.3%), and a small proportion of 6.3% in profile 1 (*N*_*profile*1_ = 59).

The three profiles were investigated based on the patterns of the three SOMEDIS-A score means (two factors and time criterion, Table 8). Moreover, a comparison of sex and a MANOVA with seven dependent variables were applied to further characterize differences between the three profiles. The MANOVA revealed a significant result [Pillai score (1, 905) = 0.32, *F*_*approx*_ (7,899) = 60.433, *p* < 0.001)]. The comparison of the three user profiles regarding sex, the SOMEDIS-A scores, the MANOVA results, as well as the variables included in the *post-hoc* MANOVA tests are presented in Table 8. Again, on the one hand, no significant differences were found regarding sex proportions, age, and the number of usage days based on the classification. On the other hand, significant differences were computed between the three profile groups with higher SMDS(-P) sum scores. The effect sizes were large for both comparisons and the differences between the SOMEDIS-A factors and time-criterion scores. The mean SOMEDIS-A, SMDS, and SMDS-P scores exceeded the cut-off values in the first profile (8). The third profile reported no prolonged problems at all (100%; 95% CI 100; 100) and the second profile consistently stated problems on single days only during the last year (100%; 95% CI 100; 100). In contrast, the first profile reported problems for longer periods (64.41%; 95% CI 52.19, 76.62) or even daily (35.59%; 95% CI 23.38, 47.81). Longer daily usage times were calculated for profile 1 than for the other groups, again with large effect sizes. The second profile users reported daily usage times to be about 50 min longer than those of the third profile users in a significant manner with a small, almost medium, effect size (0.44). Furthermore, PHQ-9 and PSS-10 sum scores were also significantly higher in profile 1 compared to the other profiles with large effect sizes. Profiles 2 and 3 also differed regarding PHQ-9 and PSS-10 scores with significantly higher values found for profile 2 and small, almost medium, effect sizes (0.46/0.49).

**Table 8:** Comparison of the three SM user profiles based on LPA.

**Variables**	**Problematic SM users (PSMU)**	**Intensive SM users (ISMU)**	**Light SM users (LSMU)**	***F*-value**	***χ^2^*/*post-hoc* Scheffé tests^***a***^**	**Cramer's V/Cohen's d**
Absolute frequency	59	543	329	–	–	–
Relative frequency in % (95%–CI)	6.34 (4.77, 7.9)	58.32 (55.16, 61.49)	35.34 (32.27, 38.41)	–	–	–
					–	–
					–	–
Female sex in % (95%–CI)	49.15 (36.4, 61.91)	50.83 (46.62, 55.03)	48.02 (42.63, 53.42)	–	0.01 NS (*p* = 0.91)	0.01
					0.00 NS (*p* = 0.99)	0.01
					0.54 NS (*p* = 0.46)	0.03
Mean SOMEDIS-A factor 1 score (SE)	9.83 (0.73)	3.15 (0.14)	0.94 (0.1)	–	–	1.91
					–	3.27
					–	0.81
Mean SOMEDIS-A factor 2 score (SE)	10.31 (0.45)	6.53 (0.13)	1.7 (0.1)	–	–	1.25
					–	4.04
					–	1.87
Mean SOMEDIS-A time criterion score (SE)	2.36 (0.06)	1 (0)	0 (0)	–	–	9.03
					–	12.59
					–	Inf
**MANOVA and** ***post-hoc*** **tests**						
Mean age (SE)	13.86 (0.24)	13.57 (0.09)	13.81 (0.13)	0.64 NS (*p* = 0.42)	–	–
Mean SMDS sum score (SE)	4.88 (0.38)	1.85 (0.08)	0.42 (0.06)	321.06^***^	−3.03^***^	1.49
					−4.46^***^	2.88
					−1.43^***^	0.86
Mean SMDS-P sum score (SE)	4.75 (0.4)	2.02 (0.1)	0.66 (0.08)	187.66^***^	−2.73^***^	1.12
					−4.09^***^	2.3
					−1.36^***^	0.66
Mean usage days per week (SE)	6.34 (0.2)	6.45 (0.06)	6.14 (0.09)	5.41^*^	0.11 NS (*p* = 0.86)	0.08
					−0.2 NS (*p* = 0.63)	0.13
					−0.2^*^	0.21
Mean time spent with SM per day [in minutes] (SE)	291.88 (30.42)	176.72 (5.44)	125.27 (5.28)	82.34^***^	−115.16^***^	0.82
					−166.61^***^	1.32
					−51.45^***^	0.44
Mean PHQ-9 sum score (SE)	10.71 (0.87)	4.76 (0.18)	2.95 (0.19)	143.65^***^	−5.95^***^	1.34
					−7.76^***^	1.87
					−1.81^***^	0.46
Mean PSS-10 sum score (SE)	21.93 (0.67)	15.91 (0.27)	12.8 (0.36)	117.53^***^	−6.02^***^	0.99
					−9.13^***^	1.46
					−3.11^***^	0.49

*** *p ≤ 0.001*,

* p ≤ 0.05, NS, not significant;

a *post-hoc tests reported in the following sequence: PSMU–ISMU, PSMU–LSMU, ISMU–LSMU. MANOVA, Multivariate Analysis of Variance; LPA, Latent Profile Analysis; SM, social media; χ^2^, chi-square; Cramér's V/Cohen's d, effect sizes; (95%–CI), 95% confidence interval; SE, standard error of the mean; SOMEDIS-A, ICD-11 Social Media Disorder Scale for Adolescents; Inf, infinite number; SMDS(-P), DSM-5 Social Media Disorder Scale (parental version); SOMEDIS-A factor 1, negative consequences; SOMEDIS-A factor 2, cognitive-behavioral symptoms; PHQ, Patient Health Questionnaire; PSS, Perceived Stress Scale*.

## Discussion

To our knowledge, this study is the first to introduce a screening instrument for assessing SMUD in adolescents according to the ICD-11 criteria for GD. SOMEDIS-A was successfully validated in a representative sample of adolescent frequent SM users and their respective parents as an instrument with good to excellent internal consistency and criterion validity as well as good to excellent discriminatory power. The instrument includes nine SMUD symptom items and one item to assess frequency and duration according to the ICD-11-time criterion. Thus, besides showing psychometrically robust properties, it is also very economical and easy to administer.

SOMEDIS-A was modified from the adolescent self-assessment instrument GADIS-A (41). The two-factorial structure of the GADIS-A was replicated for the SOMEDIS-A by an exploratory and a confirmatory factor analysis. The two factors best reflect cognitive-behavioral symptoms (such as increased SM use frequency and duration, inability to stop SM use or neglect of daily duties) and negative consequences due to SM usage behavior (such as loss of important contacts, withdrawal, poor health, or lower academic performance). The endorsed two-factor solution is at odds with approaches in which symptoms and impairments are not weighted equally, as is the case in the DSM-5 IGD definition and questionnaires derived from them, such as the SMDS (10, 11). Accordingly, functional impairment is considered by two out of nine criteria. If five of the nine criteria are met, an IGD, resp. a PSMU, can be assumed without the mandatory presence of an impairment symptom. Consequently, a differentiation between pathological and at-risk usage might not be clearly possible (76). In addition, the four-item GDT (Gaming Disorder Test) by Pontes et al. to assess ICD-11 GD favors a one-factorial solution (77). However, the equal consideration of both the behavioral SM usage pattern and the resulting negative consequences that lead to significant impairment is consistent with the biaxial model of addiction and the ICD-11 novelties (78–80). Analog to this model, impairments must be present in addition to specific symptoms to define SM use as disordered. Without meeting the impairment criterion, but with significant presence of the cognitive-behavioral symptoms, SM use could be considered hazardous (6, 7).

The internal consistency for the whole scale and the two subscales is comparable to the original GADIS-A scale with good to excellent Cronbach's α values of 0.84 to 0.91 and McDonald's ω of 0.86 to 0.93. The SOMEDIS-A sum scores positively correlated with the SMDS sum score of the DSM-5 based adolescents' self- (SMDS) and parental ratings (SMDS-P) in a strong manner. Besides good criterion reliability, excellent criterion validity is therefore indicated.

The time spent with SM (per day) positively correlated with the SOMEDIS-A sum score in a moderate manner. Hence, no significant correlation could be found with the number of SM usage days per week. Previous studies reported weak positive correlations between PSMU and usage frequencies and durations in adolescents (8, 44). In contrast, Guo et al. (81) found strong associations between PSMU and self-reported usage duration in young adults. In comparison to the cited studies, our data were acquired during the COVID-19 pandemic. A recent longitudinal study described a significant increase in the proportion of daily SM users and time spent with SM per day in German adolescents from before to during the pandemic (82). Accordingly, irrespective of the usage pattern, the majority of adolescents (75%) used SM daily during the pandemic (compared to 66% before the pandemic). The time spent with SM per day increased by about 1 h. Thus, while the usage days per week might have reached ceiling effects, the time spent with SM per day appears to be a differential measure in reference to usage patterns in a time of reduced alternative activities and contact restrictions.

Moderate positive correlations were also found between the SOMEDIS-A and the PHQ-9 sum score. These results are in line with the findings of recent systematic reviews reporting positive associations between PSMU and depression in high school students (83) and adults (35). Moreover, SOMEDIS-A as well as the PSS-10 sum scores significantly correlated in a moderate manner. Correspondingly, the meta-analytic review of Vahedi and Saiphoo (84) found small-to-medium associations between smartphone use and stress. The cross-sectional study of Beyens et al. (85) reported higher stress levels associated with SM use in adolescents. Moreover, a recent cross-sectional study on a large representative German sample of 10- to 17-year olds found a positive association between SMDS scores and psychological stress perception (19). Our results support a good criterion validity of the new scale and mirror the clinical significance of SMUD.

For a SMUD to be assumed, the cut-off values of both factors plus the time criterion had to be fulfilled. The cut-off values of the two subscales were determined by a ROC curve analysis based on the four ICD-11 associated items of the SMDS. They were slightly different from those of the GADIS-A: The value for factor 1 (negative consequences) was 6.5, one point higher, while the value for factor 2 (cognitive-behavioral symptoms) was 8.5, one point lower. Both questionnaires showed overlapping confidence intervals for the cut-off values. The confidence intervals regarding sensitivity and specificity for both cut-offs were also overlapping indicating no statistically significant differences. Slightly different cut-off values seem to be reasonable since both instruments are based on the same symptom criteria but most likely refer to separate behavioral addiction entities (86, 87). Based on the cut-offs and the time criterion, pathological SM users could be distinguished from non-pathological users. Accordingly, 3.33% (95% CI 2.18, 4.48) of the frequent SM users fulfilled the criteria of a SMUD. Keeping in mind that 92% of our initial representative adolescent sample were frequent SM users, this prevalence does not differ from the DSM-5 based estimate of 2.6% (95% CI 1.6, 3.6) in a representative sample of 12- to 17-year-old German adolescents from Germany by Wartberg et al. (88).

No differences between normal and disordered SM users were found in terms of gender. In line with our results, Wartberg et al. and Fung could also not find a significant gender influence (22, 88). In contrast, Boer et al. reported a very weak but significant positive association between female gender and PSMU (23), and van den Eijnden et al. found more boys than girls to be engaged in PSMU in one out of their three study samples (8). Diverging findings might be due to different SM definitions. In the present survey, YouTube was mentioned as an explicit example of SM since it includes a comment and like function. Whereas various SM applications seem to attract girls due to typical female usage motives (e.g., affiliation, self-disclosure), YouTube is predominantly consumed by boys (63). With respect to age, adolescents with and without SMUD also did not differ. This is consistent with the findings e.g., by van den Eijnden et al. (8, 10), and Austermann et al. (41) in comparable age groups.

Adolescents with SMUD could be clearly distinguished from other frequent users by the higher number of fulfilled DSM-5 criteria assessed by SMDS and SMDS-P, as well as by more time spent with SM. On average, adolescents classified with SMUD used SM 3 h longer per day than those without SMUD. Both groups did not differ regarding the number of usage days per week (6.39 vs. 6.33 days). In data acquired before the COVID-19 pandemic, adolescents with PSMU used SM slightly more often per week and 1 h longer than unproblematic users (44). Bányai et al. found the daily usage times of adolescents without PSMU to be 1 to 2 h lower than those of adolescents with PSMU (89). About 3 times higher PHQ-9 scores were revealed in users with SMUD compared to users without SMUD indicating more depressive symptoms in affected adolescents. According to the severity categories reported by Richardson et al. (90), the observed value of 12.71 for adolescents with SMUD refers to a moderate depressive symptom expression. With a mean score of 4.21, adolescents without SMUD did not show relevant depressive symptoms. Moreover, almost 60% higher PSS-10 scores were found for adolescents classified with SMUD compared to those without SMUD, indicating higher levels of psychological stress perception. As stress is a major predisposing factor for health problems (91), when taken together with the expression of depressive symptoms, the clinical significance of an accurate SMUD classification, is emphasized.

The results of the cut-off-based classification were supported by the LPA profile characterization. An LPA on the two SOMEDIS-A factors sum scores and the time criterion revealed three distinct profiles. Adolescents of the first profile showed significantly higher SOMEDIS-A sum scores with large effect sizes compared to the other profiles. Their factor 1 and 2 sum scores were two to three points below those of the adolescents classified as SMUD by the cut-off approach. Although it can be assumed that 3.01% of the adolescents classified in this group had a value below the cut-off of at least one factor, their mean scores were clearly above the cut-off values. Thus, they could be referred to as problematic SM users (PSMU) with a prevalence of 6.34% (95% CI 4.77, 7.9). This rate is not different from a prevalence of 5.4% reported by Boer et al. for German adolescents (23). The adolescents with PSMU had significantly higher SMDS and SMDS-P scores, more time spent with SM per day, and had larger PHQ-9 and PSS-10 scores than adolescents of the other two LPA profiles. Their mean PHQ score of 10.71 was associated with moderate depressive symptoms (90). Although subsuming pathological and at-risk SM users, this LPA group therefore features clinically relevant properties. The largest LPA group comprised 58.32% (95% CI 55.16, 61.49) of the frequent SM users. Adolescents in this profile had scores on the SOMEDIS-A, SMDS (-P), PHQ-9, and PSS-10, as well as reported time spent with SM, that were between those of the other two profiles with significant difference. Further, they used SM on average about 50 min longer per day than the third LPA group that included 35.34% (95% CI 32.27, 38.41) of the adolescents but about 115 min shorter than the PSMU group. We referred to them as intensive SM users (ISMU). Their PHQ-9 scores could be categorized as reflecting mild depressive symptoms (90). The last group was very inconspicuous in all variables surveyed suggesting no depressive symptoms and low psychological stress levels. They were referred to as light SM users (LSMU).

Collectively considering the above results, the SOMEDIS-A could be shown to be highly effective in distinguishing potentially clinically relevant from non-relevant SM users. A differentiation between light and intensive users who differ not only regarding usage patterns and durations but also subclinical depressive symptoms and stress perception could also be shown.

Although the SMUD has not yet been included in diagnostic manuals, the current results support the assumption that SMUD deserves its own conceptualization as addictive disorder (6, 9) in the context of ICD-11 behavioral addictions (68). Affected users can be typically described by criteria of established addiction concepts (9). Accordingly, SOMEDIS-A revealed usage patterns that are comparable to other (substance and behavioral) addictions (6) in a small but significant proportion of adolescents. Moreover, adolescents classified with SMUD showed greater mental distress—a common finding in patients with substance use disorders and behavioral addictions (6). In their review, Pluhar et al. described pathological media use in adolescence as a comorbidity of psychiatric conditions such as attention-deficit/hyperactivity, affective, anxiety, sleep, and autism spectrum disorders (92). However, more evidence is needed to show that SMUD is not a manifestation of another underlying pathology. Given this and the high rate of comorbidities in addiction disorders in general, a valid and reliable assessment in research and clinical settings is crucial for a better understanding of this relatively new phenomenon.

Further research on a standardized conceptualization and assessment including the two-factorial approach in SMUD should be supported to distinguish SMUD from other behavioral addictions and other mental disorders. By considering two factors involving specific symptoms and adverse outcomes, usage patterns could be described in more detail compared to polythetic approaches (80). Respectively, new hypotheses on different etiologies of pathological and hazardous SM use could be derived and tested within samples of overrepresented problematic users. More research is needed on neurobiological features of affected adolescents and the longitudinal course of the symptoms (7). Clinical validation of the SOMEDIS-A in future studies is desirable to evaluate the clinical significance of symptoms and impairments and to allow application in clinical settings.

Complementary to clinical expertise, the SOMEDIS-A could thus contribute to a better conceptualization and the early detection of potentially affected adolescents, in order to increase understanding and provide appropriate treatments and interventions as early as possible. This is urgently required by clinicians and a prerequisite for successful symptom reduction and prevention of secondary impairments, comorbidities, or even chronicity (93).

## Limitations

Although representativeness was ensured in terms of age, sex, and place of residence of the adolescent sample of frequent SM users, it may have been reduced in other respects by the data collection procedure. First, the sample only included households with sufficient knowledge of the German language, thus families with migration background might not have been sufficiently taken into account. Furthermore, about 5% of German households do not have internet access (94) and could not be considered for this study. Online questionnaires are highly valued instruments in large epidemiological surveys for economic reasons but missing data is a common problem, especially when studying parent-child dyads that include young adolescents. 110 parent-child dyads had to be excluded from further analysis, which might have further reduced representativeness. All participants were asked to answer the questionnaire independently but the influence of others cannot be ruled out. The current validation lacks objective markers such as logged usage times. The aspect of re-test reliability could not be addressed since a cross-sectional design was chosen. The present analyses were based on a categorical approach using cut-off values and neglecting behavioral spectrums. However, this approach is in line with current clinical practice, which requires efficient action even in the presence of uncertainty or “binary” yes/no decisions. Moreover, by using the four ICD-11-related items of the SMDS (13, 14) to determine cut-off values, not all relevant aspects could be covered by the criterion (e.g., loss of relationships, negative impacts on school performance or health behavior were not explicitly addressed). Most importantly, no clinical evaluation of the responses including the interpretation of the clinical relevance of the individual symptomatology exists. An external verification of the screening results by an experienced clinician would have been the gold standard for concordant validity. However, given the early stage of SMUD research, the current study supports important steps toward a better understanding of the phenomenon and early detection of affected adolescents by introducing the very first ICD-11 based screening instrument.

## Conclusion

The SOMDIS-A is the first screening tool to assess SMUD based on the ICD-11 criteria of GD. It showed good to excellent internal consistency reliability and criterion validity in a representative sample of frequent adolescent SM users. A two-factorial structure was supported analog to the original GADIS-A and in line with the biaxial model of addiction as well as the conceptual ICD-11 novelties. Accordingly, cognitive-behavioral symptoms and their negative consequences are equally weighted. The inclusion of a temporal item allows a distinction between occasional and persistent problems of clinical value. The SOMEDIS-A was able to reliably discriminate between adolescents with and without SMUD in terms of usage patterns and time spent with SM, psychological stress perception, and depressive symptoms. It is easy and economical to administer in clinical and research settings thus allowing broad application. The presented findings support the assumption that SMUD deserves its own conceptualization in the context of ICD-11 behavioral addictions and could contribute to the development of a standardized conceptualization leading to more clarity in definitions and assessment. Future clinical validation studies are warranted.

## Data Availability Statement

The raw data supporting the conclusions of this article will be made available by the corresponding author (KP) upon reasonable request after all results of the parent-child survey have been published.

## Ethics Statement

The studies involving human participants were reviewed and approved by Local Psychological Ethics Commission at the Center for Psychosocial Medicine (LPEK) of the University Medical Center Hamburg-Eppendorf (UKE). Informed consent to participate in this study was provided by the participants themselves and their legal guardian/next of kin.

## Author Contributions

KP contributed to the conceptualization, methodology, software, validation, investigation, and original draft preparation. MA contributed to the conceptualization, validation, investigation, review, editing, and visualization. RT contributed to the resources, project administration, supervision, and funding acquisition. All authors contributed to the article and approved the submitted version.

## Conflict of Interest

The authors declare that the research was conducted in the absence of any commercial or financial relationships that could be construed as a potential conflict of interest.
