# Supplementary material for: ICD-11-Based Assessment of Social Media Use Disorder in Adolescents: Development and Validation of the Social Media Use Disorder Scale for Adolescents
Source: Front Psychiatry. 2021 Apr 22;12:661483. doi: 10.3389/fpsyt.2021.661483 (PMC8100192; doi:10.3389/fpsyt.2021.661483)
Supplement: Supplementary file 1 [file Data_Sheet_1.docx]

**Appendix: Full questionnaire**

**SO**cial **ME**dia Use **DI**sorder **S**cale for **A**dolescents (**SOMEDIS-A**)

| The following statements refer to your use of social media. Thinking of the last 12 months, how strongly do you agree with the following statements? | | | | | | | |
| --- | --- | --- | --- | --- | --- | --- | --- |
| 1. **I often use social media more frequently and longer than I planned to or agreed upon with my parents.** | | | | | | | |
| strongly disagree | | disagree | partially agree/partially disagree | | agree | | strongly agree |
| 1. **I often cannot stop using social media even though it would be sensible to do so or for example my parents have told me to stop.** | | | | | | | |
| strongly disagree | | disagree | partially agree/partially disagree | | agree | | strongly agree |
| 1. **I often do not pursue interests outside the digital world (e.g., meeting friends or partner in real life, attending sports club/societies, reading books, making music) because I prefer using social media.** | | | | | | | |
| strongly disagree | | disagree | partially agree/partially disagree | | agree | | strongly agree |
| 1. **I neglect daily duties (e.g., grocery shopping, cleaning, tidying up after myself, tidying my room, obligations for school/apprenticeship/job) because I prefer using social media.** | | | | | | | |
| strongly disagree | | disagree | partially agree/partially disagree | | agree | | strongly agree |
| 1. **I often continue using social media even though it causes me stress with others (e.g., my parents, siblings, friends, partner, teachers).** | | | | | | | |
| strongly disagree | | disagree | partially agree/partially disagree | | agree | | strongly agree |
| 1. **I continue using social media although it harms my performance at school/apprenticeship/job (e.g., by being late, not participating in class, neglecting homework, worse grades).** | | | | | | | |
| strongly disagree | | disagree | partially agree/partially disagree | | agree | | strongly agree |
| 1. **Due to my social media use, I neglect my appearance, my personal hygiene, and/or my health (e.g., sleep, nutrition, exercise).** | | | | | | | |
| strongly disagree | | disagree | partially agree/partially disagree | | agree | | strongly agree |
| 1. **Due to my social media use, I risk losing important relationships (friends, family, partner) or have lost them already.** | | | | | | | |
| strongly disagree | | disagree | partially agree/partially disagree | | agree | | strongly agree |
| 1. **Due to my social media use, I have disadvantages at school/apprenticeship/job (e.g., bad [final] grades, inability to continue to the next grade/no graduation, no apprenticeship or university spot, poor reference, warning/dismissal).** | | | | | | | |
| strongly disagree | | disagree | partially agree/partially disagree | | agree | | strongly agree |
| **Now please answer the following question:** | | | | | | | |
| 1. **How often did you experience such problems, conflicts, or difficulties due to your social media use during the past year? Did this only occur on single days, during longer periods of several days to weeks or months, or was it almost daily?** | | | | | | | |
| not at all | only on single days | | | during longer periods | | almost daily | |

| **SOMEDIS-A - Evaluation** | | | | | | | | | | |
| --- | --- | --- | --- | --- | --- | --- | --- | --- | --- | --- |
| **Scoring:** | | | | | | | | | | |
| **0**  strongly diasagree | | **1**  disagree | | **2**  partially agree/  partially disagree | | | | **3**  agree | | **4**  strongly agree |
| **Factor** | **Items** | | **∑_max_** | | **Score** | | **Cut-off** | | **Cut-off is reached** | |
| cognitive behavioral symptoms | 1,2,4,5 | | 16 | |  | | >8 | | yes  no | |
| negative consequences | 3,6,7,8,9 | | 20 | |  | | >6 | | yes  no | |
| **time criterion** | **Item** | | **Response** | | | **Criterion** | | | **Criterion applies** | |
| Problem frequency | 10 | |  | | | during longer periods  *or* almost daily | | | yes  no | |

Adolescents will be screened as social media use disordered if the cut-offs for both factors are reached and the time criterion is met. Reaching the cut-off only for the factor “cognitive behavioral symptoms” might point to at-risk social media use. This indicates a higher risk for negative consequences due to the behavior. Reaching the cut-off only for the factor “negative consequences” might suggest the presence of not directly related psychological problems that should be further investigated. Reaching both cut-off values without meeting the time criterion could be an indication of at-risk use that should be further monitored.
